# Amoxicillin does not affect the development of cow’s milk allergy in a Brown Norway rat model

**DOI:** 10.1111/sji.13148

**Published:** 2022-02-28

**Authors:** Arielle Vallée Locke, Jeppe Madura Larsen, Katrine Bækby Graversen, Tine Rask Licht, Martin Iain Bahl, Katrine Lindholm Bøgh

**Affiliations:** ^1^ National Food Institute Technical University of Denmark Kgs. Lyngby Denmark

**Keywords:** antibiotics, food allergy, gut microbiota

## Abstract

The use of antibiotics as well as changes in the gut microbiota have been linked to development of food allergy in childhood. It remains unknown whether administration of a single clinically relevant antibiotic directly promotes food allergy development when administrated during the sensitisation phase in an experimental animal model. We investigated whether the antibiotic amoxicillin affected gut microbiota composition, development of cow's milk allergy (CMA) and frequencies of allergic effector cells and regulatory T cells in the intestine. Brown Norway rats were given daily oral gavages of amoxicillin for six weeks and whey protein concentrate (WPC) with or without cholera toxin three times per week for the last five weeks. Microbiota composition in faeces and small intestine was analysed by 16S rRNA sequencing. The development of CMA was assessed by WPC‐specific IgE in serum, ear swelling response to WPC and body hypothermia following oral gavage of WPC. Allergic effector cells were analysed by histology, and frequencies of regulatory and activated T cells were analysed by flow cytometry. Amoxicillin administration reduced faecal microbiota diversity, reduced the relative abundance of Firmicutes and increased the abundance of Bacteroidetes and Proteobacteria. Despite these effects, amoxicillin did not affect the development of CMA, nor the frequencies of allergic effector cells or regulatory T cells. Thus, amoxicillin does not carry a direct risk for food allergy development when administrated in an experimental model of allergic sensitisation to WPC via the gut. This finding suggests that confounding factors may better explain the epidemiological link between antibiotic use and food allergy.

**Abbreviations**
AMX: amoxicillin
ANCOM: analysis of composition of microbiomes
ANOSIM: analysis of similarities
ASV: amplicon sequence variant
BLG: β‐lactoglobulin
BN: Brown Norway
CMA: cow's milk allergy
CT: cholera toxin
CTR: control
DADA2: Divisive Amplicon Denoising Algorithm 2
ELISA: enzyme‐linked immunosorbant assay
EPI: epithelium; EST, ear swelling test
FoxP3: forkhead box protein 3
HE: haematoxylin and eosin
HRP: horseradish peroxidase
LP: lamina propria
mLN: mesenteric lymph nodes
OC: oral challenge
PAS: periodic acid‐Schiff
PBS: phosphate‐buffered saline
PCoA: principal coordinate analysis
PERMDISP: permutational analysis of multivariate dispersions
PP: Peyer's patches
QIIME: Quantitative Insights Into Microbiota Ecology
RT: room temperature
SI: small intestine
TB: toluidine Blue
Th: T helper
TMB: 3,3’, 5,5’‐tetramethylbenzidine
Treg: regulatory T cell
WPC: whey protein concentrate

## 1 INTRODUCTION

Food allergy is a growing health problem in terms of incidence, prevalence, persistency and severity—particularly in the Western world.^1^ Currently, 6%‐8% of children are affected by food allergy.^2^ Cow's milk is an important nutrient in small children's diet. However, cow's milk contains proteins with high allergenicity, which can give rise to allergic sensitization and allergen‐specific IgE‐mediated adverse reactions in the gastrointestinal tract, skin, respiratory system and systemic anaphylactic symptoms.^3^ The prevalence of cow's milk allergy (CMA) varies with age and country, but has been reported in 2%‐7% of small children and 0.1%‐0.5% of adults in developed countries.^4^ Thus, while most children outgrow their CMA, about 15% retain their allergy into adulthood.^3^, ^5^

The aetiology of food allergy remains largely unknown, but the disease is believed to arise from the complex interaction between genetic and environmental risk factors. Several environmental factors have been reported to modify the risk for food allergy, including urbanization, infant formula feeding, consumption of processed foods, antibiotic use, delivery by caesarean section and decreased incidence of infections.^1^, ^6^, ^7^ The recently formulated epithelial barrier hypothesis proposes that these environmental exposures affect epithelial integrity leading to increased allergy either directly due to increased permeability to allergens and/or due to changes in microbiota composition leading to bacterial translocation and tissue microinflammation.^8^, ^9^ The risk of food allergy development is affected by factors that may modify the gut microbiota composition, which in turn may shape disease risk by affecting gut immune function. Indeed, food allergy is associated with changes in gut microbiota composition,^10^, ^11^ and specific gut bacteria have been shown to protect against development of food allergy in experimental models.^11^, ^12^ Antibiotics are potent modifiers of the bacterial microbiota, and the use of these compounds has been associated with the risk of developing food allergy and other atopic diseases in epidemiological studies.^13^, ^14^, ^15^, ^16^ However, further delineation of the causal role and mechanistic basis of antibiotics in food allergy development requires the use of animal models. Previous studies found that antibiotic treatment promoted allergic sensitization to peanut in mouse models of peanut allergy.^17^, ^18^ These studies used a mixture of different antibiotics, and one study reported the eradication of almost all bacteria except for *Lactobacillaceae*.^17^ Thus, there is a need to further investigate the role of antibiotics in food allergy development by focusing on the administration of single clinically relevant antibiotic. Amoxicillin is a broad‐spectrum beta‐lactam antibiotic and is the most frequently prescribed antibiotic in the primary care setting.^19^ The WHO reports that amoxicillin is the most frequently used oral antibiotic worldwide,^20^ and the American Centers for Disease Control and Prevention recommends amoxicillin as the first‐in‐line therapy for common paediatric infections,^21^ including acute sinusitis, acute otitis media and pharyngitis. The main adverse reaction to amoxicillin treatment is diarrhoea,^22^ which likely can be attributed to the disturbance of the gut microbiota composition in humans.^23^, ^24^ We previously found that amoxicillin did not affect the development of oral tolerance to cow's milk whey protein concentrate (WPC) in a Brown Norway (BN) rat model of CMA.^25^ This finding indicates that this particular antibiotic does not promote food allergy by inhibiting homeostatic tolerance induction in the gut. Here, we investigate how amoxicillin affects the development of CMA using a model with cholera toxin (CT)‐mediated intragastric sensitization to WPC in allergy‐prone BN rats.

## 2 MATERIALS AND METHODS

### 2.1 Animals

BN rats were from the in‐house breeding colony, the National Food Institute, Technical University of Denmark. Rats were bred and raised for ≥10 generations on an in‐house produced milk‐free diet based on rice flour, potato protein and fish meal, as previously described,^26^ with the exception of maize flakes being substituted with rice flour. Diet and water were given ad libitum. Rats of same gender were housed two by two in makrolon cages with a 12‐hour light/dark cycle, at a temperature of 22 ± 1 C°, and with a relative humidity of 55 ± 5%. Rats were observed twice a day. Ethics approval for the animal experiment was given by the Danish Animal Experiments Inspectorate with the authorization number 2015‐15‐0201‐00553‐C1. The experiment was overseen by the Institute's Animal Welfare Committee for animal care and use.

### 2.2 Animal experiment

**FIGURE 1:**
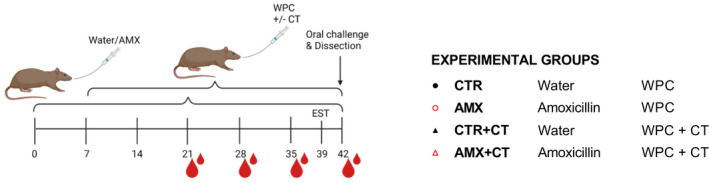
Outline of animal experiment. Allergy‐prone Brown Norway rats were given daily administrations of water as control (CTR) or amoxicillin (AMX) by gavage for 6 weeks (starting Day 0). After the first week, cow's milk whey protein concentrate (WPC) was administrated with or without cholera toxin (CT) by gavage three times per week (starting Day 7). Animals were subjected to an ear swelling test (EST) using WPC (Day 39) and an oral challenge using WPC by gavage (Day 42). Animals were sacrificed and dissected for collection of tissue and faecal samples (Day 42). The figure is created with BioRender.com

Female BN rats with an age of 3.5‐5 weeks were allocated into four groups of 12 rats (Figure 1) for the induction of CMA using a CT‐mediated model.^27^ Half of the rats were gavaged daily with 30 mg of amoxicillin (AMX) in 0.5 mL sterile Milli Q water and the other half with 0.5 mL sterile Milli Q water alone (CTR) for a total of 6 weeks. Starting on Day 7, the oral gavage solutions additionally included 10 mg WPC without (CTR and AMX) or with (CTR+CT and AMX+CT) 20 μg of CT three times per week for 5 weeks. The dose and duration of AMX administration were based on our previous studies in BN rats.^25^, ^28^ In order to include rats with a uniform age, the experiment was divided into two separate sets of experiments without (CTR and AMX groups) and with (CTR+CT and AMX+CT groups) CT respectively.

WPC was kindly provided by Arla Foods Ingredients, Videbæk, Denmark, while CT was purchased from List Biological Laboratories Inc (cat. no. 100B, Cambell, CA, US).

Blood samples were collected on Days 21, 28 and 35 by sublingual vein puncture and allowed to coagulate (1 hour at room temperature (RT) followed by overnight at 4°C) before serum was obtained after centrifugation (1800*g*, 20 min, RT) and stored at −20°C until antibody analysis.

An ear swelling test^29^ was performed by intradermal injections of 10 μg WPC in 20 µL PBS (137 mmol/L NaCl (cat. no. 1.06404.1000, VWR, Radnor, PA, US), 2.7 mmol/L KCl (cat. no. P9333, Sigma‐Aldrich, St. Louis, MO, US), 10 mmol/L Na_2_HPO_4_ (cat. no. 1.06580.1000, Merck, Darmstadt, Germany), 1.8 mmol/L KH_2_PO_4_ (cat. no. 153184 U, VWR), pH 7.2) using a BD Micro‐Fine syringe and needle (30G, cat. no. U‐100, BD, Franklin Lakes, NJ, US) on day 39 under hypnorm‐dormicum (Glostrup Apotek, Glostrup, Denmark) anaesthesia as previously described.^25^ Ear swelling was calculated as the change in ear thickness measured at the same spot before and one hour after injection using a Digital Micrometer (range 0‐25 mm, resolution 0.001 mm, accuracy ±0.002 mm; cat no. 705‐1279, RS PRO, Corby, UK).

### 2.3 Dissection

On Day 42, rats were challenged by oral gavage of 0.5 mL PBS with 100 mg WPC, and the body temperature was recorded after 15 min using a rectal thermocouple meter (Digi Sense, Aarhus, Denmark). Subsequently, rats were sacrificed by exsanguination using carbon dioxide as anaesthesia, and blood was drawn and processed for serum, as described above, for antibody analysis. Additional blood was collected in sodium‐heparin coated tubes (cat no. 367 869, BD) for analysis by flow cytometry.

During the dissection on Day 42, a faecal pellet (closest to the rectum) and small intestine (SI) content (squeezed from the first 20 cm SI obtained after removing the first 7 cm from the stomach), samples were collected and stored at −80°C until preparation for microbiota analysis using 16S rRNA gene sequencing. A second faecal pellet (second closest to the rectum) was collected for IgA extraction using 1 mL cold PBS with 0.05% (w/v) NaN_3_ (cat. no. S2002, Sigma‐Aldrich) per 100 mg sample in a bead beater MM300 (Retsch GmbH, Haan, Germany) for 15 min at 30 cycles/s followed by centrifugation at 16,000*g* at 4°C for 10 min and stored at −20°C until analysis.

Intestinal epithelium (EPI), lamina propria (LP) and Peyer's patches (PP) were collected after the intestine was flushed using saline (0.9% (w/v) NaCl in sterile water), to remove luminal content, for analysis of intestinal β‐lactoglobulin (BLG) uptake. Mesenteric lymph nodes (mLN), PP and SI tissues were collected for flow cytometry, and SI and colon tissue samples were fixed overnight in 4% (v/v) paraformaldehyde for histological analyses as previously described.^28^, ^30^

### 2.4 Microbiota analysis by 16S rRNA‐sequencing

Faecal and SI bacterial microbiota compositions were analysed by 16S rRNA amplicon sequencing, as previously described.^28^ Briefly, DNA was extracted from intestinal samples using the DNeasy PowerLyzer PowerSoil Kit (cat. no. 12855‐100, Qiagen, Hilden, Germany) according to the manufacturer's instruction and stored at −20°C until analysis. The V3‐region of the 16S rRNA gene was amplified from each sample separately with barcoded primers, purified and mixed equally to generate sequencing libraries. The Ion Torrent PGM platform (Life Technologies, Carlsbad, CA, US) was used for sequencing as previously described.^28^, ^31^ Raw sequence reads were processed using the CLC bio genomic workbench (Qiagen, Hilden, Germany) in order to de‐multiplex, remove sequencing primers and perform quality trimming using default settings. Further quality trimming was performed in Divisive Amplicon Denoising Algorithm 2 (DADA2, Version 1.10.1) using default settings as described elsewhere.^31^ Finally, amplicon sequence variants (ASVs) were identified and taxonomically classified using the Ribosomal Database Project Multi‐classifier tool.^32^ Microbial α‐ and β‐diversity metrics and relative abundance estimates at different taxonomical levels were calculated by running the Quantitative Insights Into Microbiota Ecology (QIIME) diversity core‐metrics‐phylogenetic script based on a rooted phylogenetic tree (QIIME2 Version 2019.1). To eliminate bias from uneven sampling depth, samples were rarefied to 5,000 and 3,000 reads per sample for faecal and SI samples respectively. Faecal samples with less than 5,000 reads per sample and SI samples with less than 3,000 reads per sample were discarded from the analysis.

### 2.5 ELISAs

WPC‐specific IgG1 antibody titres were determined by an indirect ELISA, as previously described.^30^, ^33^ Briefly, plates (96 well Plates, Maxisorp, Nunc, cat. no. 10547781, Roskilde, Denmark) were coated with WPC and specific antibodies were detected using horseradish peroxidase (HRP)‐labelled mouse‐α‐rat IgG1 (clone G17E7, cat. no. 3060‐05, Southern Biotech, Birmingham, AL, US). WPC‐specific IgG2a, IgG2b and IgG2c antibody titres were determined in a similar manner using HRP‐labelled goat‐α‐rat IgG2a (cat. no. PA1‐84709, Thermo Fisher Scientific, Middletown, VA, US), goat‐α‐rat IgG2b (cat. no. PA1‐84710, Thermo Fisher Scientific), IgG2b and goat‐α‐rat IgG2c (cat. no. PA1‐84711, Thermo Fisher Scientific), respectively.

WPC‐specific IgE antibody titres were determined by IgE‐capture ELISA, as previously described.^30^, ^33^ Briefly, plates were coated with mouse‐α‐rat IgE (clone MARE‐1, cat. no. HDMAB‐123, HybriDomus, Cytotech, Hellebæk, Denmark) and blocked with rabbit serum (cat. no. S2500‐500, Biowest, Nuaillé, France). Specific antibodies were detected using digoxigenin‐coupled WPC and HRP‐coupled sheep‐α‐digoxigenin (cat. no. 11633716001, Roche Diagnostics GmbH, Mannheim, Germany).

Total IgA antibody titres were determined by IgA‐sandwich ELISA, as previously described.^28^ Briefly, plates were coated with mouse‐α‐rat IgA (clone MARA‐1, cat. no. MCA191, BioRad, Oxford, UK) and blocked with egg white protein (cat.no E0500, Sigma‐Aldrich). Antibodies were detected using HRP‐conjugated goat anti‐rat IgA (cat. no. STAR111P, BioRad, Oxford, UK).

ELISAs were developed using 3,3′,5,5‐tetramethylbenzidine (TMB)‐one as substrate (cat. no. 4380A, Kem‐En‐Tec, Taastrup, Denmark) and stopped with sulfuric acid. Absorbance was measured at 450 nm with a reference wavelength of 630 nm using a microtitre reader (Gen5, BioTek Instruments, Winooski, VT, US). Antibody titres were expressed as Log_2_ titre values. All plates included positive and negative control samples to ensure consistent assay performance.

### 2.6 Intestinal protein uptake

Total proteins were extracted from tissue samples using 10 µL tris‐lysis buffer (150 mM NaCl (cat. no. 1.06404.1000, VWR), 20 mM Tris (cat. no. A1087, AppliChem GmbH, Darmstadt, Germany), 1mM EGTA (cat. no. E3889, Sigma‐Aldrich), 1% (v/v) IGEPAL (v/v; cat. no. 18896, Sigma‐Aldrich) and 1 mM EDTA (cat. no. 03690, Sigma‐Aldrich)) with 2% Halt protease inhibitor cocktail (v/v) (cat. no. 78438, Thermo Fisher Scientific) per mg tissue. Samples were homogenised using stainless steel beads (Qiagen, Hilden, Germany) and the TissueLyser II (Qiagen) at 30 cycles/s for 2 min. Samples were incubated on ice for 20 min and mixed by vortexing every 5 min. Samples were centrifuged at 15,000*g* for 20 min at 4°C, and the supernatants were stored at −80°C until analysis. The whey protein BLG were quantified in tissue extracts using a commercial bovine BLG ELISA kit (cat. no. A10‐125A, Bethyl Laboratories, Montgomery, AL, US) according to the manufacturer's protocol with the exception that plates were coated overnight.

### 2.7 Histology

Fixated tissues were dehydrated in an ethanol gradient of 77%‐99% (absolute ethanol, cat. no. 83813.360, VWR), cleared using xylene (cat. no. 28973.363, VWR) and embedded in paraffin (cat. no. 2270.60.60, Hounisen, Skanderborg, Denmark). Sections of 2 μm were stained with Meyer's Hematoxylin (cat. no. AMPQ00254.0500, Ampliqon, Odense, Denmark) and Eosin Y (cat. no. 341973R, VWR) to identify eosinophils and 0.5% Toluidine Blue (TB; cat. no. 89640, Sigma‐Aldrich) in 1 M hydrochloric acid to identify mast cells, or Periodic acid‐Schiff (PAS; periodic acid: Cat. no. 1.00524.0025, Merck and Schiff′s reagent: Cat. 3952016, Sigma‐Aldrich) to identify goblet cells.^34^ Slides were examined using a Leica DMR upright microscope (Leica Microsystems GmbH, Wetzlar, Germany). The software ImagePro Plus 7.0 (MediaCybernetics, Rockville, MD, US) was used for image analysis. Villus length was measured from the villus tip to the crypt‐villus junction. Cell count and villi length in the SI were averaged from three sections of three similar consecutive villi and crypts. Cell count in the colon was averaged from six individual crypts. Analysis of histological sections was performed blinded.

### 2.8 Flow cytometry

Single‐cell suspensions were prepared from tissue samples and analysed by flow cytometry, as previously described.^28^ Briefly, one million cells were blocked with 10% (v/v) rat serum (produced in‐house) and anti‐CD32 (clone D34‐485, cat.no. 550273, BD) in FACS buffer (2% (v/v) foetal bovine serum (cat. no. F9665, Sigma‐Aldrich) and 0.05% (w/v) NaN_3_ in PBS), and subsequently stained using the following antibody cocktail: anti‐CD3/PerCp‐eF710 (clone eBioG4.18, cat. no. 44‐0030‐82, Thermo Fisher Scientific), anti‐CD4/PE‐Cy7 (clone OX‐35, cat. no. 561578, BD), anti‐CD45/APC‐eF780 (clone OX‐1, cat. no. 47‐0461‐82, Thermo Fisher Scientific), anti‐B220/PE (clone HIS24, cat. no. 12‐0460‐82, Thermo Fisher Scientific) and anti‐CD25/BV421 (clone OX‐39, cat. no. 565608, BD). Cells were subsequently stained intracellularly by anti‐Foxp3/FITC (clone JFK‐16s, cat. no. 11‐5773‐82, Thermo Fisher Scientific) using the BD Transcription Factor buffer set (cat. no. 562574, BD) according to manufacturer's recommendations. Blood samples were diluted twofold in FACS buffer, surface‐stained as above, lysed using VersaLyse (cat. no. A09777, Beckman Coulter, Copenhagen, Denmark) and stained intracellularly as described above. Data were acquired on a BD FACS Canto II (BD) or BD LSRFortessa system (BD) and analysed using FlowJo version 10.4.2 (BD).

### 2.9 Statistics

Differences in α‐diversity indices (Shannon index) between groups were tested with the Kruskal‐Wallis pairwise test through QIIME2. Differences in β‐diversity between groups were assessed by applying analysis of similarities (ANOSIM) to weighted and unweighted UniFrac distances, and within‐group dispersion was analysed by permutational analysis of multivariate dispersions (PERMDISP) both in QIIME2.^35^ Differential abundance/mean relative abundance of the genera between groups in both faecal and SI samples was determined by analysis of composition of microbiomes (ANCOM) in R.^36^ CT‐mediated changes in the microbiota were not analysed across the two sets of experiments.

The remaining data were analysed using the Kruskal‐Wallis test followed by Dunn's post hoc test comparing the following groups: CTR vs. AMX, CTR+CT vs. AMX+CT, CTR vs. CTR+CT and AMX vs. AMX+CT, using GraphPad Prism version 8.1.1 (GraphPad Software, California, US). Correlational analyses were performed using the non‐parametric Spearman R correlation in Graphpad Prism. Statistically significant differences are indicated by asterisks: *P ≤ .05; **P ≤ .01; ***P ≤ .001; ****P ≤ .0001.

## 3 RESULTS

### 3.1 Intestinal microbiota composition

**FIGURE 2:**
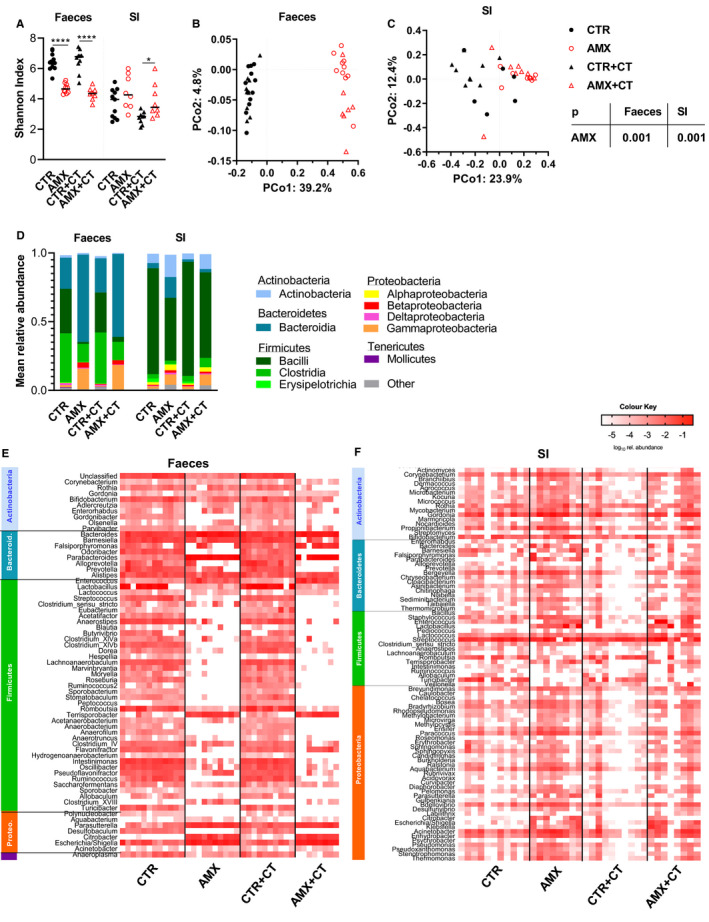
Faecal and small intestine (SI) microbiota composition by 16S rRNA gene sequencing. Shannon α‐diversity index for faecal and SI microbiota samples in water control (CTR, filled black circles), amoxicillin (AMX, open red circles), water control with cholera toxin (CTR+CT, filled black triangles) or amoxicillin with cholera toxin (AMX+CT, open red triangles) groups of rats (A; *n* = 8‐12 per group). Each symbol represents a single rat and horizontal lines indicate median values. Principal coordinate analysis (PCoA) plot of weighted UniFrac distances representing the β‐diversity in the faecal (B; *n* = 7‐12 per group) and SI (C; *n* = 6‐9 per group) microbiota samples. The relative abundances of bacterial phyla in faeces and SI (D). Mean relative abundance of bacterial genera in the faecal (E; *n* = 8‐12 per group) and SI (F; *n* = 8‐12 per group) microbiota samples. Colours indicate abundance from low abundant genera in white to high abundant genera in red. Heatmaps were constructed based on the relative abundance of the genera present in ≥35% of the samples

The 6‐week daily amoxicillin administration regimen (Figure 1) significantly reduced faecal microbiota α‐diversity (Figure 2A), while it was found to slightly increase SI microbiota α‐diversity (Figure 2A). Amoxicillin significantly affected β‐diversity in both faeces and the SI microbiota (Figure 2B‐C). Effects on faecal microbiota were characterised by a higher relative abundance of Bacteroidetes and Proteobacteria (Gamma‐ and Betaproteobacteria), and a lower relative abundance of Firmicutes (Bacilli and Clostridia) in the amoxicillin administered rats (Figure 2D). Similar amoxicillin‐induced effects were observed in the SI microbiota (Figure 2D). Amoxicillin affected multiple genera within the Bacteroidetes, Firmicutes and Proteobacteria phyla in the faecal microbiota (Figure 2E), but only showed minor effects at the genera level in the SI (Figure 2F).

### 3.2 Development of cow's milk allergy

**FIGURE 3:**
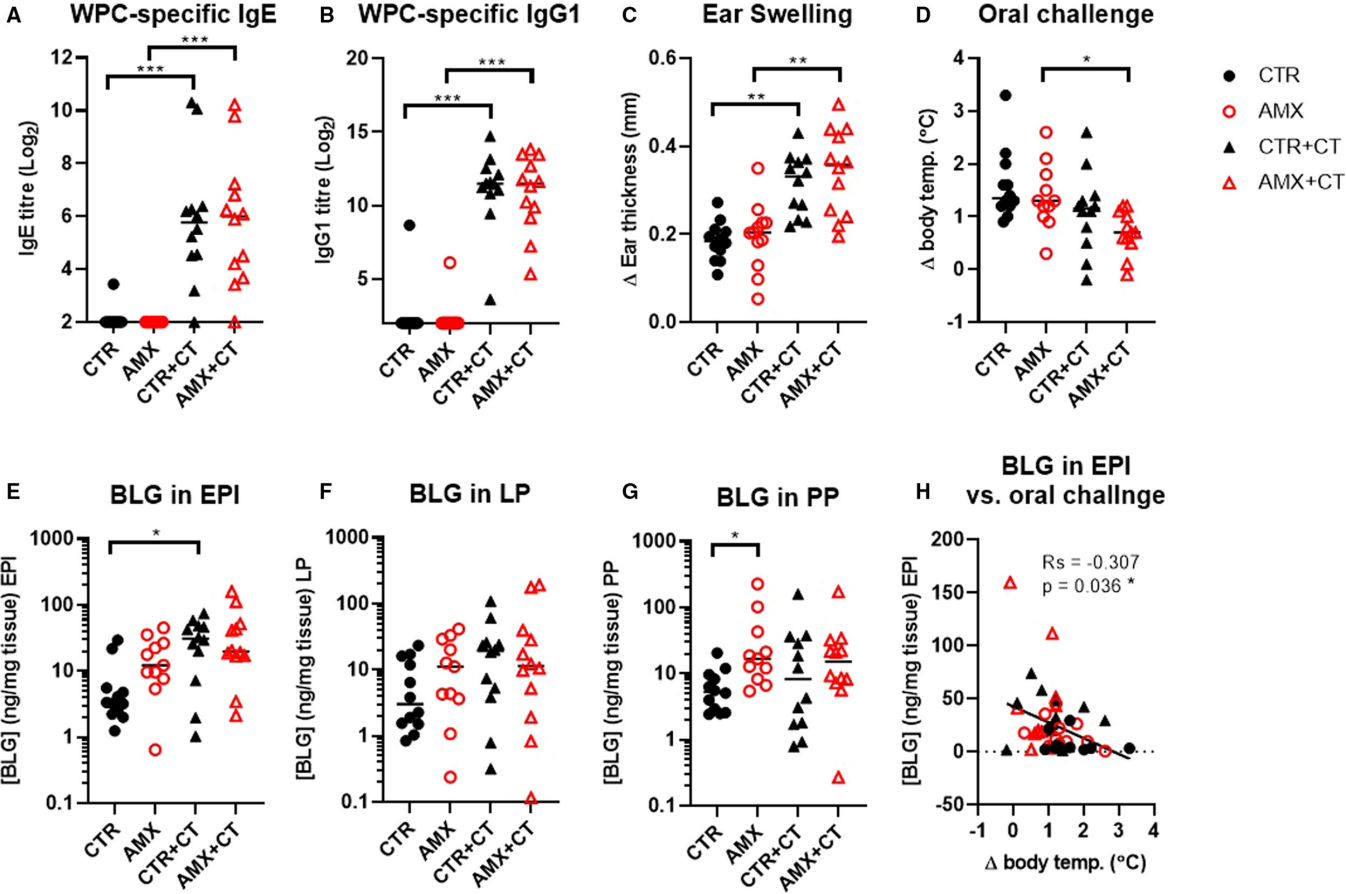
Allergic sensitisation, ear swelling response, oral challenge and intestinal antigen uptake. WPC‐specific IgE titres in serum A, WPC‐specific IgG1 titres in serum B, clinical reactivity to WPC by ear swelling test C, and change in core body temperature following oral WPC challenge D, in water control (CTR, filled black circles), amoxicillin (AMX, open red circles), water control with cholera toxin (CTR+CT, filled black triangles) or amoxicillin with cholera toxin (AMX+CT, open red triangles) groups of rats. Quantification of β‐lactoglobulin (BLG) in intestinal epithelial (EPI), lamina propria (LP) and Peyer's patches (PP) 15 min after oral WPC challenge (E‐F). Correlation between BLG concentration in EPI and changes in core body temperature after oral challenge (Spearman correlation; H). Each symbol represents a single rat and horizontal lines indicate median values (*n* = 11‐12 per group)

Administration of WPC without CT did not induce sensitisation (CTR and AMX, Figure 3A), whereas administration together with CT induced sensitisation to WPC, as demonstrated by the induction of WPC‐specific IgE in serum (CTR+CT and AMX+CT, Figure 3A). However, concomitant daily administration with amoxicillin did not increase IgE level to WPC when analysed at the day of sacrifice (CTR vs. AMX and CTR+CT vs. AMX+CT, Figure 3A), nor earlier during the sensitisation regime (Supporting Information, Figure S1A‐C). Similarly, only administration of WPC together with CT induced WPC‐specific IgG1 (CTR+CT and AMX+CT, Figure 3B). CT administration was also found to promote WPC‐specific IgG2a, IgG2 and IgG2c levels, which were unaffected by the amoxicillin administration (Supporting Information, Figure S1D‐F).

Allergic sensitisation to WPC was associated with clinical reactivity to intradermally injected WPC, demonstrating the development of functional IgE (CTR+CT and AMX+CT, Figure 3C), yet the amoxicillin administration was not found to aggravate this reaction (CTR+CT vs. AMX+CT, Figure 3C). Oral challenge with WPC had only a minor effect on core body temperature, indicating that this model represents low anaphylactogenic CMA (Figure 3D).

### 3.3 Intestinal whey protein uptake

Post‐challenge BLG content in EPI, LP and PP was assessed to evaluate intestinal antigen uptake (Figure 3E‐G). The results indicated that CT administration increased the amount of BLG in the epithelial layer, either as a direct effect of the adjuvant capacity of CT or as an indirect effect of the CT‐induced allergic sensitisation (Figure 3E). Furthermore, the results indicated that amoxicillin increased the amount of BLG in the PP (Figure 3G). The amount of BLG in the EPI inversely correlated with the core body temperature following oral challenge (Figure 3H), indicating that this route of antigen uptake is related to the elicitation of the allergic response.

### 3.4 Intestinal allergic inflammation

**FIGURE 4:**
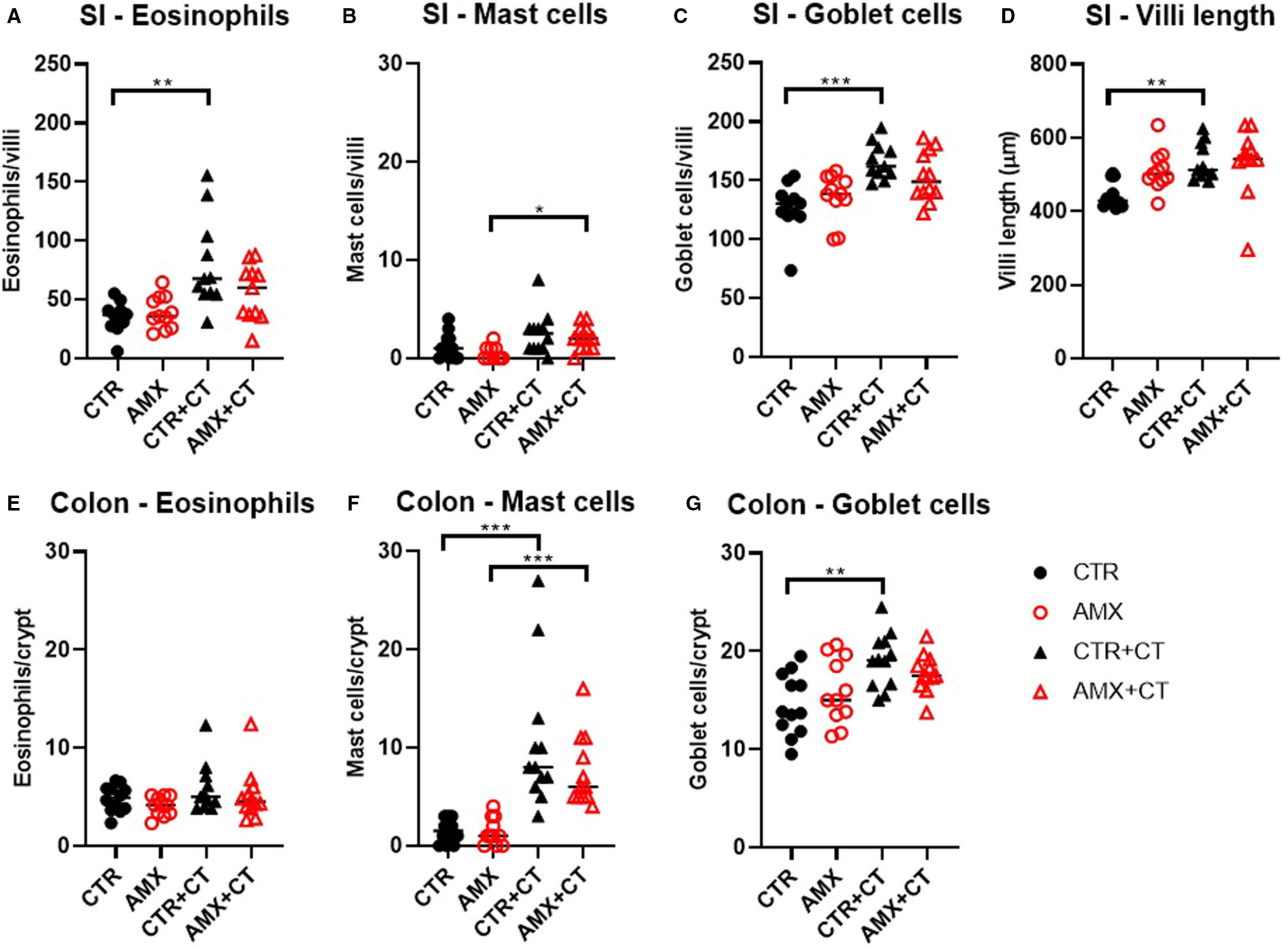
Allergic effector cells and villi morphology in the intestine. Eosinophils (A + E), mast cells (B + F) or goblet cells (C + G) frequencies in the small intestine (SI) and in colon identified by histology using haematoxylin‐eosin, toluidine blue and periodic acid‐Schiff staining, respectively, for water control (CTR, filled black circles), amoxicillin (AMX, open red circles), water control with cholera toxin (CTR+CT, filled black triangles) or amoxicillin with cholera toxin (AMX+CT, open red triangles) groups of rats. Average villi length in the SI (D). Each symbol represents a single rat and horizontal lines indicate median values (*n* = 10‐12 per group)

The frequency of allergic effector cells was analysed in intestinal tissues by histology. Allergic sensitisation to WPC induced by CT administration was associated with increased numbers of eosinophils, mast cells and goblet cells in the SI (Figure 4A‐C). The frequency of mast cells and goblet cells was also increased in the colon of sensitised rats (Figure 4E‐G). Furthermore, the allergic inflammation was associated with increased villi length in the SI (Figure 4D). Administration of amoxicillin was not found to affect the number of allergic effector cells in the SI and colon (CTR vs. AMX, Figure 4A‐G), nor did it affect the allergic inflammation mediated by CT‐induced WPC sensitisation (CTR+CT vs. AMX+CT, Figure 4A‐G).

### 3.5 Intestinal immune regulation and activation

**FIGURE 5:**
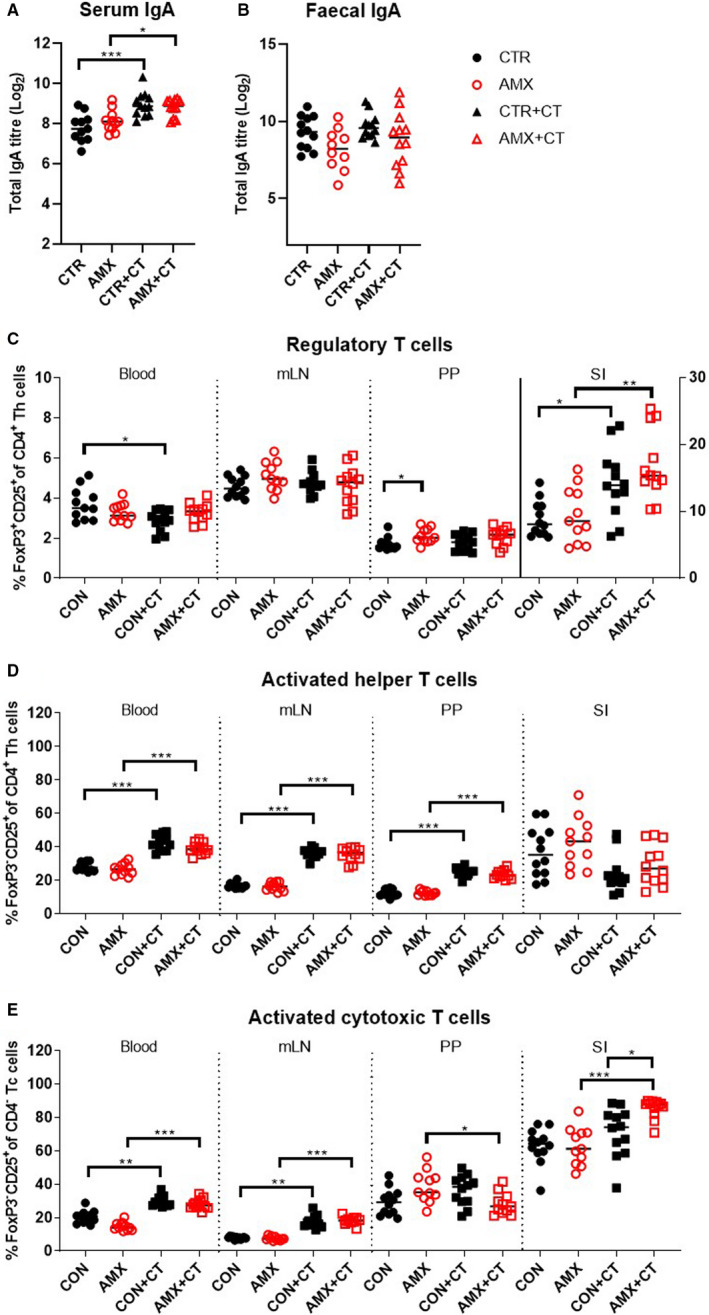
Immune regulatory responses in the intestine. Total IgA levels in serum A, and faecal B, samples after sacrifice for water control (CTR, filled black circles), amoxicillin (AMX, open red circles), water control with cholera toxin (CTR+CT, filled black triangles) or amoxicillin with cholera toxin (AMX+CT, open red triangles) groups of rats. Frequencies of FoxP3+CD25+ regulatory T cells C, FoxP3‐CD25+ activated helper T cells D, and FoxP3‐CD25+ activated cytotoxic T cells E. Each symbol represents a single rat and horizontal lines indicate median values (*n* = 10‐12 per group)

Regulatory T cells (Tregs) and IgA‐mediated immune responses are central in maintaining intestinal homeostasis and modulating the commensal microbiota. CT‐induced WPC sensitisation was associated with increased total IgA levels in serum, but not in faecal samples (Figure 5A‐B). Amoxicillin administration was not found to affect IgA levels in these compartments. Neither did amoxicillin affect the frequency of Treg cells (CD4^+^FoxP3^+^CD25^+^), activated helper T cells (CD4^+^FoxP3^‐^CD25^+^) or activated cytotoxic T cells (CD4^‐^FoxP3^‐^CD25^+^) in the blood or intestine (Figure 5C‐E). However, CT‐induced WPC sensitisation was associated with significant T cell activation (Figure 5C‐E). Please refer to Supporting Information, Figure S2 for gating strategy and representative plots from the analysis of blood, mLN, PP and SI tissue samples.

## 4 DISCUSSION

The 6‐week long‐term amoxicillin administration was found to decrease faecal microbiota diversity while slightly increasing the microbiota diversity within the SI of the BN rats. Amoxicillin decreased the relative abundance of predominantly Clostridia and Bacilli in the faecal microbiota, and Bacilli in the SI microbiota. This decrease in Firmicutes was accompanied by the expansion of Gammaproteobacteria within both the faecal and SI microbiotas. These findings are in line with our previous study of a 1‐week short‐term amoxicillin administration in BN rats reporting similar effects on the microbiota.^28^ This indicates that amoxicillin‐induced changes in microbiota composition occur rapidly and persist with continued amoxicillin administration. Importantly, the effects of amoxicillin on the microbiota of BN rats are comparable to previous studies in humans,^37^, ^38^, ^39^, ^40^ supporting the translational relevance of our model.

The association between changes in gut microbiota composition and allergic disease is well‐documented,^41^, ^42^ yet there are currently no clear‐cut associations between bacterial members of the gut microbiota and disease.^41^ This may be due to differences between specific atopic diseases (food allergy, asthma, rhinitis etc), disease endotypes, study methodology and timing (eg before vs. after disease onset).^41^ However, recent studies in food allergic infants found that the absence of specific clostridial species was associated with disease.^11^, ^12^ Administration of specific Clostridiales or Bacteroidales consortia has been demonstrated to protect from food allergy development in a mouse model.^12^, ^17^ The underlying mechanism of Clostridiales‐mediated protection from food allergy was found to be the expansion of intestinal Treg cells via MyD88‐dependend signals working directly on the Treg cells.^12^ The importance of Clostridiales in promoting immune regulatory mechanisms in the gut is supported by previous studies in mice demonstrating the expansion of Treg cells in the gut and protection from experimental colitis.^43^ In the present study, we found that amoxicillin administration caused a decrease in faecal Clostridiales, but this decrease was not associated with a decrease in the frequency of Treg cells nor the promotion of food allergy development. This finding suggests that amoxicillin administration does not mediate sufficient eradication of Clostridiales to potentiate food allergy development in our model. In comparison, previous studies demonstrated that treatment using a mixture of aminoglycoside (kanamycin and gentamicin), polymixin (colistin), glycopeptide (vancomycin) and nitroimidazole (metronidazole) antibiotics promoted allergic sensitization to peanut in a mouse model of peanut allergy.^17^, ^18^ However, this antibiotic mixture was reported to lead to the eradication of almost all bacteria except for Lactobacillaceae.^17^ In this context, the complete eradication of Clostridiales suggests a central role of these bacteria in homeostatic tolerance in the gut. However, administering a mixture of antibiotics does not reflect the typical use of antibiotics in the clinic. Thus, we here studied the role of a single commonly used antibiotic.

Despite effects on gut microbiota composition, amoxicillin did not promote sensitisation to WPC or affect the clinical response to WPC in animals sensitised to WPC. The frequency of allergic effector cells in the SI and colon was not only increased in sensitised rats, but was also not affected by amoxicillin administration. Additionally, amoxicillin did not affect the frequency of classical FoxP3^+^CD25^+^ Treg cells in the blood, mLN, PP or SI. We previously reported that amoxicillin promotes classical FoxP3^+^CD25^+^ Treg cells in the SI, but not in the blood, mLN or PP after a short 1‐week regimen of daily administrations in BN rats.^28^ However, this effect seems only to be transient, as amoxicillin did not affect the overall frequency of classical FoxP3^+^CD25^+^ Tregs in the intestine five weeks after a 4‐week regimen of daily administrations in BN rats.^25^ Combined, the findings indicate that amoxicillin has limited long‐term effects on homeostatic immune regulation and risk for food allergy development in the gut.

Our findings suggest that amoxicillin does not possess an acute risk for food allergy development when administrated in an experimental setting driving allergic sensitisation to cow's milk whey proteins via the gut. Yet, it is increasingly appreciated that the skin plays a central role in the sensitisation to food.^44^, ^45^, ^46^ A line of research has shown that enrofloxacin affects cutaneous immune responses to haptens and ovalbumin.^47^, ^48^, ^49^ Interestingly, perinatal exposure to enrofloxacin, but not exposure in adult mice, promotes allergic immune responses.^48^, ^49^ These studies suggest that antibiotic use may promote allergy development during a specific window of opportunity. Indeed, the microbiota is known to affect early immune development, and changes in the microbiota at this stage could lead to increased risk of disease due to alterations in the immune system.^50^

Epidemiological studies indicate that different antibiotics confer distinct risks for food allergy development,^13^, ^15^ suggesting that either different mechanisms may be at play for each class of antibiotics, or that underlying risk factors for various infections carry their own independent risks for food allergy. Interestingly, antibiotic use by the mother before or during pregnancy, or by the offspring, are all risk factors for food allergy in the offspring,^13^ suggesting a heritable confounding factor for antibiotic use and food allergy. Similar observations have been indicated for other atopic diseases, including atopic dermatitis and asthma.^51^, ^52^ These observations support the hypothesis that the link between antibiotic use and allergic disease risk may be confounded by underlying immunological risk factors. We speculate that confounding immunological risk factors could arise in the general population from antibiotics use over many generations leading to population‐wide changes in the commensal microbiota (ie loss of allergy‐protective commensal bacteria, as propose by the ‘old friends’ hypothesis^53^). Furthermore, even microbiota‐unrelated environmental factors could drive the concomitant need for antibiotic use and allergy development via lifestyle‐related exposures that directly affect immune function. There is a need to further explore these hypotheses using appropriate translational models.

## CONFLICT OF INTEREST

The authors declare no competing interests with regard to publication of this manuscript.

## AUTHOR CONTRIBUTIONS

KLB, MIB and TRL conceived and designed the study. AVL and KBG conducted the animal studies. AVL performed the laboratory work under supervision of KBG, KLB, MIB and JML. AVL, JML and KBG analysed and presented the data. JML and AVL drafted the manuscript, and KBG and KLB revised it. All authors made substantial intellectual contributions, reviewed the manuscript critically and approved the final version of the manuscript.

## ETHICS STATEMENT

Ethics approval for the animal experiment was given by the Danish Animal Experiments Inspectorate with the authorization number 2015‐15‐0201‐00553‐C1. The experiment was overseen by the Institute's Animal Welfare Committee for animal care and use.

## Supporting information

Supplementary Material

## Data Availability

The 16S rRNA gene sequence data have been deposited in the NCBI Sequence Read Archive under BioProject number PRJNA755147. Other data that support the findings of this study are available from the corresponding author upon reasonable request.
