# Supplementary material for: Amoxicillin does not affect the development of cow’s milk allergy in a Brown Norway rat model
Source: Scand J Immunol. 2022 Feb 28;95(5):e13148. doi: 10.1111/sji.13148 (PMC9285443; doi:10.1111/sji.13148)
Supplement: Supplementary file 1 — Supplementary Material [file SJI-95-0-s001.docx]

**SUPPORTING INFORMATION**

**
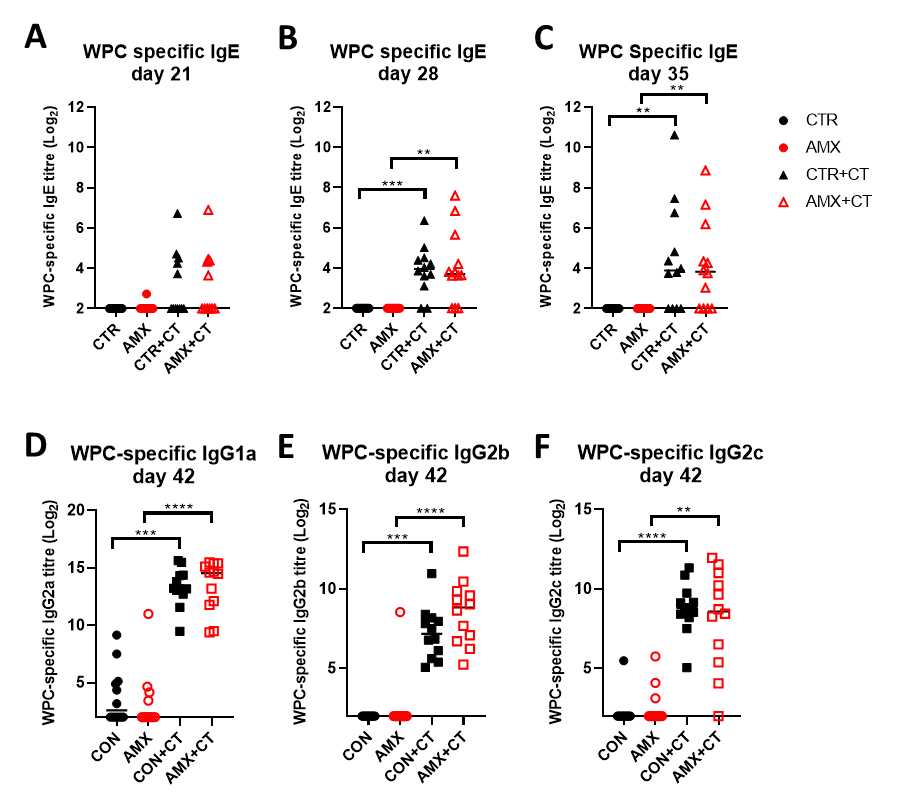
**

**Figure S1.** **Antibody-responses to WPC.** Cow’s milk whey protein concentrate (WPC)-specific IgE titres in serum on day 21 (A), 28 (B), and 35 (C), and WPC-specific IgG2a (D), IgG2b (E) and IgG2c (F) in serum on day 42 in water control (CTR, filled black circles), amoxicillin (AMX, open red circles), water control with cholera toxin (CTR+CT, filled black triangles), or amoxicillin with cholera toxin (AMX+CT, open red triangles) groups of rats. Each symbol represents a single rat and horizontal lines indicate median values.

**
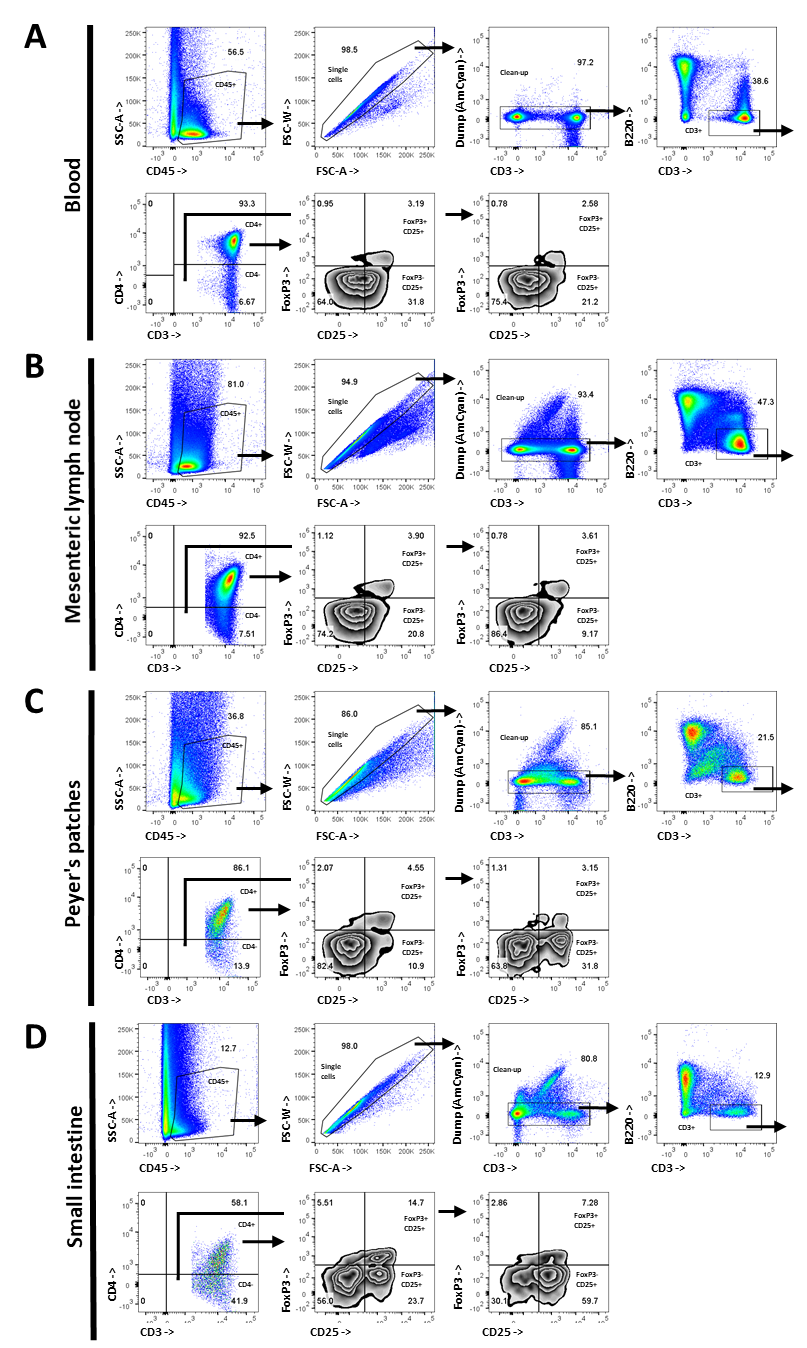
**

**Figure S2.** **Flow cytometry gating strategy.** Gating for the identification of regulatory T cells (CD4+FoxP3+CD25+), and activated helper T cells (CD4+FoxP3-CD25+) and activated cytotoxic T cells (CD4-FoxP3-CD25+) in blood (A), mesenteric lymph nodes (B), Peyer's patches (C), and small intestine (D).
